# Estimating Costs of Market Exclusivity Extensions For 4 Top-Selling Prescription Drugs in the US

**DOI:** 10.1001/jamahealthforum.2025.2631

**Published:** 2025-08-22

**Authors:** Dongzhe Hong, S. Sean Tu, Reed F. Beall, Massimiliano Russo, Benjamin N. Rome, Aaron S. Kesselheim, Ameet Sarpatwari

**Affiliations:** 1 Program On Regulation, Therapeutics, and Law (PORTAL), Division of Pharmacoepidemiology and Pharmacoeconomics, Brigham and Women’s Hospital, Boston, Massachusetts; 2 Harvard Medical School, Boston, Massachusetts; 3 University of Alabama School of Law, Tuscaloosa; 4 Department of Community Health Sciences, Cumming School of Medicine, University of Calgary, Calgary, Alberta, Canada; 5 Department of Statistics, The Ohio State University, Columbus

## Abstract

**Importance:**

Brand-name drugs in the US are sold at high prices during market exclusivity periods defined by their patents, before prices are lowered by generic competition. Drug manufacturers use several strategies to extend these market exclusivity periods and delay generic competition, including obtaining overlapping thickets of patents.

**Objective:**

To estimate excess US spending associated with delays in generic competition due to extended market exclusivity for 4 top-selling drugs.

**Design, Setting, and Participants:**

This retrospective serial cross-sectional study focused on 4 top-selling drugs that experienced new generic competition between 2014 and 2018 to allow enough time for determining postexclusivity price trajectories: imatinib (Gleevec, cancer), glatiramer (Copaxone, multiple sclerosis), celecoxib (Celebrex, arthritis), and bimatoprost (Lumigan, glaucoma). Drug monthly spending data from 2011 to 2021 were retrieved from a large commercial claims database (Merative MarketScan) and a random sample of Medicare beneficiaries with at least 1 month of Medicare Parts A, B, and D coverage and adjusted for estimated rebates obtained from SSR Health, LLC. The analysis was performed between March 2023 and January 2024.

**Exposures:**

Extended market exclusivity was calculated as the time between expiration of the key patent and first generic marketing.

**Main Outcomes and Measures:**

The primary outcome was net monthly national drug spending in commercial insurance and Medicare Part D. Spending was estimated under 2 scenarios: (1) the status quo, reflecting observed spending trends, and (2) a counterfactual scenario, modeling spending in the absence of extended market exclusivity. Segmented linear regression analyses were used to assess level and slope changes in monthly spending following generic entry. Weights were applied to extrapolate sample-based estimates to the full US commercially insured and Medicare Part D populations.

**Results:**

Market exclusivity extensions beyond expiration of the key patent ranged from 7 (celecoxib) to 13 (glatiramer) months. In the absence of extended market exclusivity, and over a 2-year period following generic competition, net spending would have decreased by $3.5 billion, including $1.9 (95% CI, $1.3-$2.5) billion in commercial plans and $1.6 (95% CI, $1.1-$2.1) billion in Medicare—including $67 (95% CI, $22-$115) million for bimatoprost, $726 (95% CI, $516-$938) million for celecoxib, $1.7 (95% CI, $1.0-$2.4) billion for glatiramer, and $1.0 (95% CI, $0.8-$1.2) billion for imatinib.

**Conclusions and Relevance:**

This study found that promoting timely generic availability and avoiding extending market exclusivity for top-selling drugs may result in substantial savings for US patients and payers, including both public and private health insurance programs.

## Key Points

**Question:**

What is the excess US spending associated with delays in generic competition due to extended market exclusivity?

**Findings:**

In this retrospective serial cross-sectional analysis of 4 top-selling drugs (imatinib, glatiramer, celecoxib, and bimatoprost) including 5.7 million Medicare beneficiaries, extended market exclusivity beyond key patent expirations ranged from 7 to 13 months. This delay resulted in an estimated $3.5 billion in excess spending over a 2-year period, with $1.9 billion in commercial plans and $1.6 billion in Medicare.

**Meaning:**

These findings indicate that policies promoting timely generic drug availability by limiting market exclusivity extensions could lead to substantial savings for US patients and payers.

## Teaser

This cross-sectional study estimates excess US spending associated with delays in generic competition due to extended market exclusivity for 4 top-selling brand-name drugs that experienced new generic competition between 2014 and 2018.

## Introduction

Individuals in the US spend more per capita on pharmaceuticals than patients in other high-income countries.^1^ Between 2011 and 2020, inflation-adjusted net US spending on prescription drugs increased 37%, from $263 billion to $359 billion, the equivalent of about $1100 per person.^2^ Brand-name drugs accounted for 80% of prescription drug spending, which brand-name manufacturers sell at high prices during patent-protected periods of market exclusivity that usually last around 12 to 14 years from the launch of the drug.^3,4,5^ The only market factor that has been shown to substantially lower spending on prescription drugs in the US is the introduction of Food and Drug Administration (FDA)–approved generic drugs, which are often automatically substituted for brand-name products by pharmacists, leading to price competition. Generic substitution can vary across states, with some mandating substitution unless specified otherwise by the prescriber, while others require patient consent before substitution.^6^

Lack of timely generic competition contributes to high US drug spending. An important factor that determines the duration of a brand-name drug’s market exclusivity is the patent on a drug’s underlying active ingredient, which is also often the strongest patent (ie, broadest and least likely to be overturned in court). Manufacturers frequently choose their active ingredient patent for patent term extension, a process through which drug manufacturers can get up to 5 additional years of patent protection to account for clinical testing and regulatory review time.^7,8,9,10,11^ In addition, for successful drug products, manufacturers regularly build up large portfolios of nonactive ingredient patents, which might cover the drug’s formulation, mechanism of delivery, and method of manufacture, or by introducing modified versions of drugs with new patented formulations, mechanisms of delivery, and methods of manufacture.^12,13,14,15^ Patent extensions and secondary patents can complicate generic entry.

Previous studies have examined the extent and costs of delayed generic entry. An analysis of the 106 top selling drugs between 2005 and 2015 found 76 (72%) had their “patent cliff”—the last expiration date of a set of patents—extended at least once, with 54 (51%) receiving multiple extensions.^16^ Another investigation found the 12 highest selling drugs in 2017 secured exclusivity protections averaging 38 years—nearly double the standard duration of a single drug patent.^17^ Generic entry for 31 of 69 (45%) brand-name drugs predicted to lose market exclusivity between 2010 and 2016 was delayed by more than a quarter of a year, resulting in $761 million in excess costs to Medicaid alone.^18^

To obtain systemwide estimations of the cost of delayed generic entry, we sought to estimate national excess spending associated with market exclusivity extensions for 4 top-selling drugs. We focused on costs to Medicare and commercial payers, which make up most prescription drug spending in the US.

## Methods

### Study Design and Data Sources

We conducted a retrospective serial cross-sectional study using data from 2 health insurance claims databases (Merative MarketScan [MarketScan] and a Medicare sample), 1 drug classification database (First Databank), 1 patent database (annual editions of the FDA’s *Approved Drug Products with Therapeutic Equivalence Evaluations* [Orange Book] through 2022), and 1 pricing database (SSR Health Database). MarketScan provided longitudinal claims (2011-2021) from a national sample of approximately 30 million commercially insured people at any given time. The Medicare sample captured longitudinal claims (2013-2018) on 5.7 million randomly selected beneficiaries with at least 1 month of Medicare Parts A, B, and D coverage. First Databank included national drug codes (NDCs) for all FDA-approved drugs, and the Orange Book contained manufacturer-reported patents on all FDA-approved small-molecule drugs. Finally, the SSR Health Database provided estimations of quarterly net price (net of rebates and other price concessions) and sales information for prescription drugs manufactured by publicly owned companies. The institutional review board at Mass General Brigham granted approval with a waiver of informed consent to use deidentified claims data. Results were reported in accordance with the Strengthening the Reporting of Observational Studies in Epidemiology (STROBE) reporting guidelines.^19^

### Study Cohort

We identified brand-name drugs that faced generic competition between 2014 and 2018. We excluded those without continuous net price data in the SSR Health Database during their exclusivity period or a key patent in the FDA’s Orange Book. To focus on drugs with standard approval pathways, we included only those approved as new molecular entities or new active ingredients, and to ensure the availability of spending data, we removed drugs without at least 1.5 years of data before and after generic entry in claims databases or those with key patents expiring before 2013. Finally, we excluded drugs without an extended market exclusivity period, resulting in a final sample of 4 drugs: the cancer drug imatinib (Gleevec), the multiple sclerosis drug glatiramer (Copaxone), the arthritis drug celecoxib (Celebrex), and the glaucoma drug bimatoprost (Lumigan) (eFigure in Supplement 1).

### Original and Extended Market Exclusivity Periods

**Figure 1.**
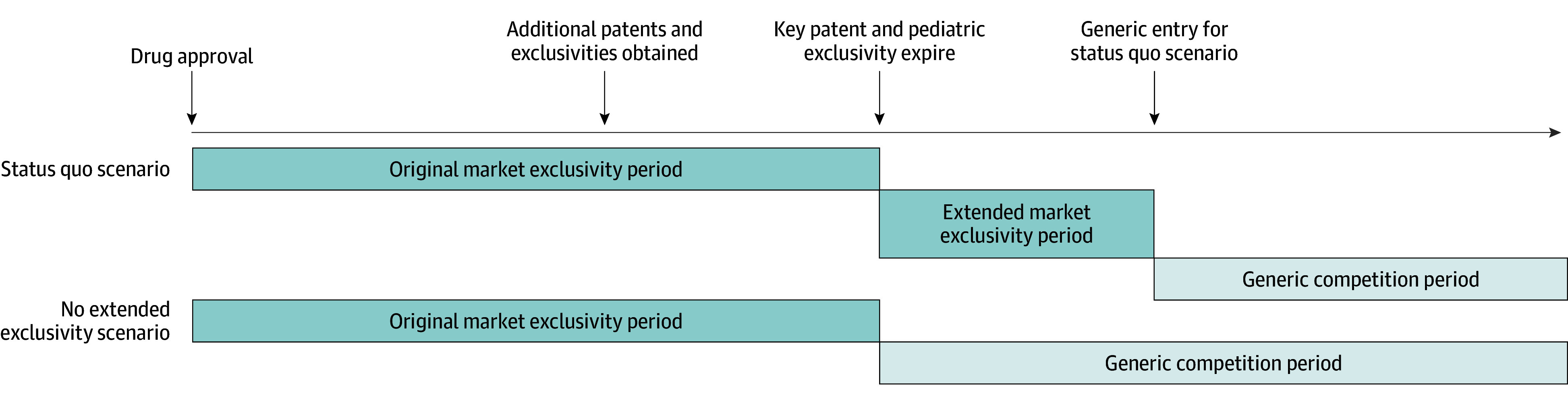
Market Exclusivity Periods Relative to Patent and Generic Entry Milestones The status quo scenario is the observed scenario in which existing patent and exclusivities do not change. The no extended exclusivity scenario is the hypothetical scenario in which the extended market exclusivity period is removed and replaced with the generic competition period.

We identified the originally expected and actual market exclusivity periods for each drug. As in prior studies, the originally expected market exclusivity period was defined as the time between regulatory approval of the drug and expiration of the drug’s key patent: the composition of matter patent covering the drug’s underlying active ingredient or another patent chosen by the manufacturer to receive a patent term extension (Figure 1; eMethods 1 in Supplement 1).^18,20^ This term was calculated as inclusive of any patent term extension and pediatric exclusivity, a 6-month extension of existing patents awarded for fulfilling an FDA request to test the drug in children. The actual market exclusivity period included the time from original FDA approval to the month of first generic marketing. So-called authorized generics, drugs marketed by the brand-name manufacturer under a generic label,^21^ were excluded from consideration of first generic marketing dates.

### Outcomes

#### Database-Specific Net Drug Spending and Prescription Counts

We calculated net monthly spending on and the number of prescriptions for the 4 study drugs in MarketScan and the Medicare sample. First, we used the identified drug product NDCs to determine gross monthly spending on brand-name and generic versions of each drug in the 2 databases (eMethods 2 in Supplement 1). Second, we estimated net spending by applying quarterly non-Medicaid discount rates from the SSR Health Database to the gross monthly brand-name spending for each drug within that quarter. Because major rebates and other price concessions are rarely offered for generic drugs in Medicare or commercial plans, we assumed that gross generic drug spending was equivalent to net generic drug spending. All spending totals were adjusted to 2022 US dollars using the Consumer Price Index for all urban consumers.

#### National Excess Spending

To estimate net spending on the 4 drugs among the full commercially insured and Medicare populations in the US, we applied weights to account for differences between our study sample and the total insured population. MarketScan captures a subset of commercially insured individuals, and our Medicare sample represents a subset of Medicare Part D beneficiaries. To scale our sample-based estimates to national spending levels, we calculated weights as the ratio of (1) the total number of commercially insured individuals in the US to those in MarketScan, and (2) the number of Medicare Part D beneficiaries nationwide to those in our Medicare sample (eMethods 3 in Supplement 1).^22,23^ By applying these weights, we adjusted for variations in sample composition over time, ensuring that our estimates reflect national spending patterns. The same weights were used in the status quo scenario and counterfactual scenarios to maintain comparability. For the primary analysis, national excess spending was estimated over the 2-year postextended exclusivity period. We further examined the dynamic effects of extended market exclusivity by estimating incremental and cumulative excess spending over the 1-year, 2-year, and 3-year postextended exclusivity periods.

### Statistical Analysis

We used segmented linear regression analyses to assess changes in both spending and prescription fills following generic entry. The unit of analysis was monthly drug spending and monthly number of prescriptions. For each drug, we used the Interrupted Time Series Analysis (ITSA) package in Stata statistical software, using generalized linear model regression to assess immediate level changes (abrupt changes in monthly spending and number of prescriptions at generic entry) and slope changes (changes in the trends of monthly spending and number of prescriptions after generic entry). Spending was log-transformed, whereas the number of prescriptions was analyzed without transformation. We tested for autocorrelation using the Cumby-Huizinga test in each model to detect serial correlation. If significant autocorrelation was present (2-sided *P* < .05), we incorporated autoregressive terms to adjust for the highest significant lag identified. The appropriate lag structure was determined separately for each drug and specified in the final regression models.^24^

Significant changes in the level or slope of each outcome were determined based on a 2-sided *P* < .05. Data collection was performed using SAS statistical software (version 9.4; SAS Institute, Inc), and analyses were conducted using STATA (version 17; StataCorp). To compute 95% CIs for the national excess spending, we used parametric bootstrap approach coded in R (version 4.2.1, R Foundation). Full model specifications and specific steps to estimate national excess spending are provided in eMethods 4 in Supplement 1. The analysis was performed between March 2023 and January 2024.

## Results

The 4 drugs in the study—imatinib, glatiramer, celecoxib, and bimatoprost (Table 1; eTable 1 in Supplement 1)—were originally approved by the FDA between 1996 (glatiramer) and 2001 (imatinib, celecoxib), and market exclusivity ranged from 14 years (bimatoprost) to 18 years (glatiramer). Net US sales in the year before first generic entry ranged from $307 million (bimatoprost) to $3.1 billion (glatiramer). Extended market exclusivity ranged from 7 months (celecoxib and imatinib) to 13 months (glatiramer).

**Table 1.** Study Drug Characteristics^a^

Drug name	Original indications	Original approval date	Key patent and pediatric exclusivity expiration date	US sales in year prior to generic entry, $ millions	Generic entry date	Total market exclusivity, y	Extended market exclusivity, mo
Lumigan (Bimatoprost)	Glaucoma	March 16, 2001	August 20, 2014	307	May 2015	14	9
Celebrex (Celecoxib)	Osteoarthritis and rheumatoid arthritis	December 31, 1998	May 30, 2014	1933	December 2014	16	7
Copaxone (Glatiramer)	Multiple sclerosis	December 20, 1996	May 24, 2014	3113	June 2015	18	13
Gleevec (Imatinib)	Chronic myeloid leukemia and gastrointestinal stromal tumors	May 10, 2001	July 4, 2015	2533	February 2016	15	7

^a^ Original indications and approval dates were obtained from the Food and Drug Administration (FDA) drug labels. Key patent expiration and exclusivity data were sourced from the FDA Orange Book. Annual sales in the year prior to generic entry were obtained from SSR Health LLC. Generic entry dates were determined based on the first observed claims data in MarketScan and the Medicare sample.

### Spending Changes After Generic Competition

In MarketScan (eTable 2 in Supplement 1), estimated monthly spending levels immediately after generic entry were 93% of pre-entry levels for imatinib (95% CI, 87%-100%; *P* < .001), 113% for glatiramer (95% CI, 103%-124%; *P* = .008), 60% for celecoxib (95% CI, 53%-68%; *P* < .001), and 91% for bimatoprost (95% CI, 80%-103%; *P* = .13), relative to the month before generic entry. The monthly spending slopes were estimated at 98% of pre-entry trends for imatinib (95% CI, 98%-98%; *P* < .001), 98% for glatiramer (95% CI, 98%-98%; *P* < .001), 98% for celecoxib (95% CI, 98%-98%; *P* < .001), and 100% for bimatoprost (95% CI, 99%-100%; *P* < .001).

In the Medicare sample (eTable 3 in Supplement 1), estimated monthly spending levels immediately after generic entry were 85% of pre-entry levels for imatinib (95% CI, 79%-92%; *P* < .001), 100% for glatiramer (95% CI, 94%-106%; *P* = .81), 87% for celecoxib (95% CI, 81%-94%; *P* = .001), and 103% for bimatoprost (95% CI, 98%-108%; *P* = .21), relative to the month before generic entry. The monthly spending slopes were estimated at 99% of pre-entry trends for imatinib (95% CI, 98%-100%; *P* < .001), 96% for celecoxib (95% CI, 95%-96%; *P* < .001), and 99% for bimatoprost (95% CI, 99%-100%; *P* = .009). Detailed model fit statistics are provided in eTable 4 in Supplement 1.

### Number of Prescriptions Changes After Generic Competition

In MarketScan (eTable 5 in Supplement 1), the number of prescriptions declined immediately after generic entry for celecoxib (−6307.99; 95% CI, −11 203.68 to −1412.30; *P* = .01) and bimatoprost (−4031.80, 95% CI, −6020.93 to −2042.67; *P* < .001), whereas no significant level changes were observed for imatinib or glatiramer. After generic entry, the trend in prescriptions per month increased for imatinib (13.30; 95% CI, 10.87-15.74; *P* < .001), glatiramer (46.13; 95% CI, 31.28-60.97; *P* < .001), celecoxib (745.91; 95% CI, 601.31-890.52; *P* < .001), and bimatoprost (180.26; 95% CI, 115.79-244.74; *P* < .001).

In the Medicare sample (eTable 6 in Supplement 1), the number of prescriptions for glatiramer increased immediately after generic entry (297.15; 95% CI, 167.50-426.80; *P* < .001), whereas those for celecoxib decreased immediately after generic entry (−2193.81; 95% CI, −3570.79 to −816.82; *P* = .002). After generic entry, the trend in prescriptions per month decreased for imatinib (−7.66; 95% CI, −9.60 to −5.71; *P* < .001), glatiramer (−15.50; 95% CI, −21.64 to −9.37; *P* < .001), and bimatoprost (−122.47; 95% CI, −178.07 to −66.87; *P* < .001), but increased for celecoxib (179.28; 95% CI, 89.59-268.97; *P* < .001).

### Excess Spending

If generic entry had occurred when originally expected, drug-specific spending over the remaining months of market exclusivity and 2 years following the actual start of generic competition for the drugs would have decreased between $3.4 million (bimatoprost, $63.5 million to $60.1 million) and $195 million (glatiramer, $1527 million to $1332 million) in MarketScan, and between $6.1 million (bimatoprost, $81.3 million to $75.2 million) and $82 million (imatinib, $560 million to $478 million) in the Medicare sample. Between 1 and 3 years after extended market exclusivity, total savings in the MarketScan sample varied from $245 million to $457 million, and total savings in the Medicare sample varied from $102 million to $293 million (eTables 7-9 in Supplement 1).

### National Excess Net Spending

After applying weights, net national spending would have been $3.5 (95% CI, $2.7-$4.3) billion lower had generic competition begun after expiration of the key patent for the 4 drugs, including $1.9 (95% CI, $1.3-$ 2.5) billion in commercial plans and $1.6 (95% CI, $1.1-$2.1) billion in Medicare (Table 2). Total national excess spending would have been $1.7 (95% CI, $1.0-$2.4) billion for glatiramer, followed by $1.0 (95% CI, $0.8-$1.2) billion for imatinib, $726 (95% CI, $516-$938) million for celecoxib, and $67 (95% CI, $22-$115) million for bimatoprost.

**Table 2.** National Excess Spending Associated With Extended Market Exclusivity^a^

Drug	Estimated national spending, $ (95% CI), millions								
	National commercial			National Medicare				National estimates, total excess spending	
	With extended market exclusivity	Without extended market exclusivity	Excess spending	With extended market exclusivity	Without extended market exclusivity	Excess spending		
Gleevec (Imatinib)	4399 (4179-4630)	3978 (3778-4188)	421 (293-561)	3310 (3126-3511)	2723 (2533-2932)	586 (406-769)	1007 (787-1238)	
Copaxone (Glatiramer)	7603 (7159-8069)	6550 (6032-7138)	1053 (499-1606)	4667 (4463-4891)	3979 (3609-4400)	687 (228-1115	1740 (1029-2436)	
Celebrex (Celecoxib)	1671 (1540-1808)	1261 (1139-1393)	410 (280-545)	1680 (1580-1790)	1364 (1232-1515)	316 (143-480)	726 (516-938)	
Lumigan (Bimatoprost)	320 (302-340)	304 (283-327)	16 (−2 to 36)	678 (654-706)	627 (587-669)	51 (10-95)	67 (22-115)	
Total	13 993 (13 483-14 520)	12 093 (11 515-12 711)	1900 (1311-2490)	10 335 (10 048-10 651)	8694 (8252-9191)	1641 (1128-2132)	3540 (2774-4323)	

^a^ Estimated national spending was derived from MarketScan and Medicare claims data and modeled using segmented regression analysis, as described in the Methods section. Spending estimates were scaled to national levels using analytic weights to adjust for differences between the study sample and the total insured population. Excess spending was calculated by comparing estimated spending under the status quo scenario (extended exclusivity remains in place) with a counterfactual scenario assuming generic entry at the key patent expiration date.

**Figure 2.**
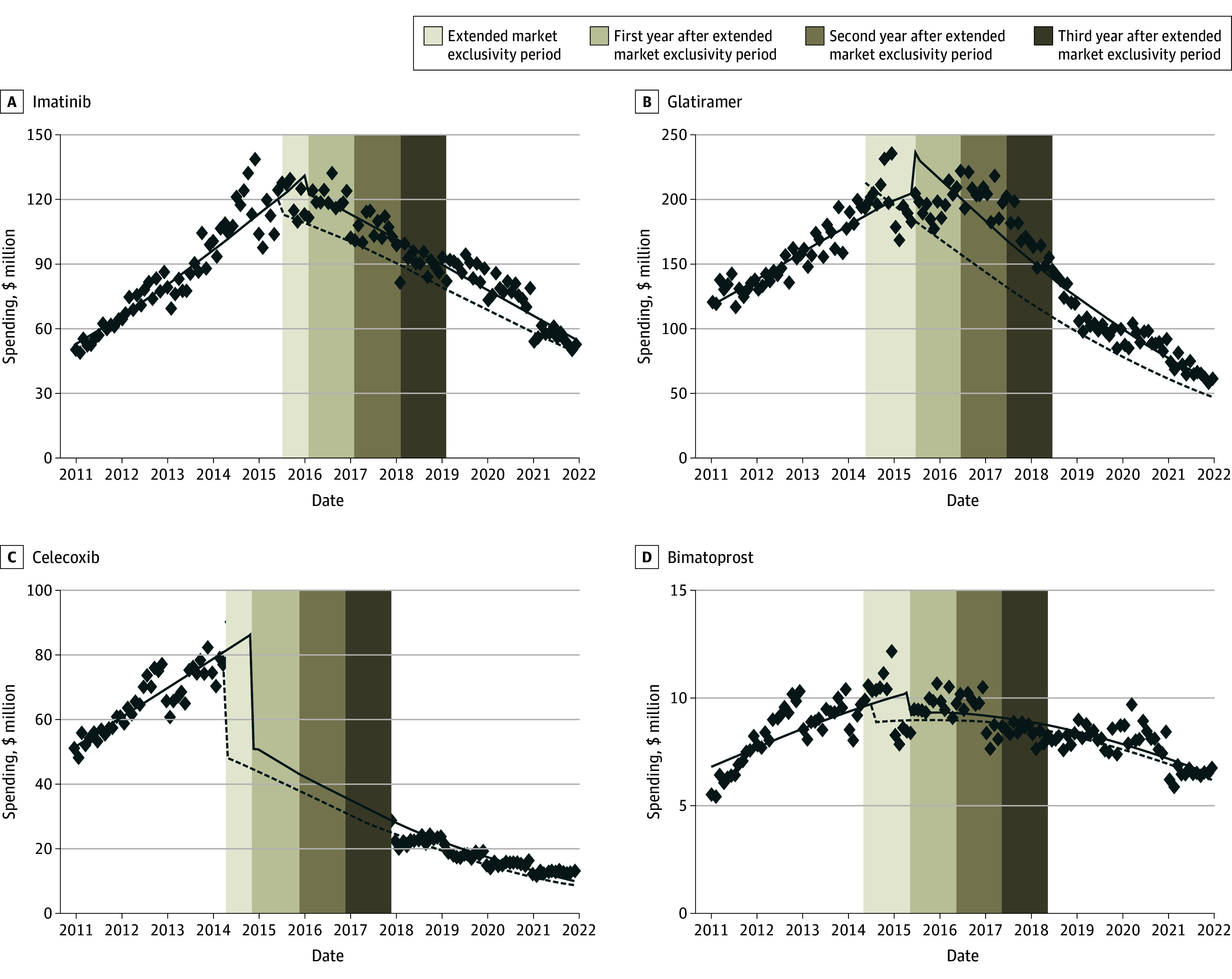
Estimated National Monthly Drug Spending With or Without Extended Market Exclusivity in Commercial Plans The estimates were weighted to extrapolate the database spending estimates from the MarketScan sample to national levels, ensuring generalizability to the full commercially insured population. The solid line shows the estimated monthly spending of each drug in the status quo scenario. The dashed line shows the estimated monthly spending of each drug in no extended exclusivity period. The data points are the actual monthly net spending. The area between the solid line and dashed line within the vertical area shows the excess spending of each drug in different time periods. Vertical axes are different for each drug. The excess spending is visually represented by the area between the solid and dashed lines within the shaded vertical areas.

**Figure 3.**
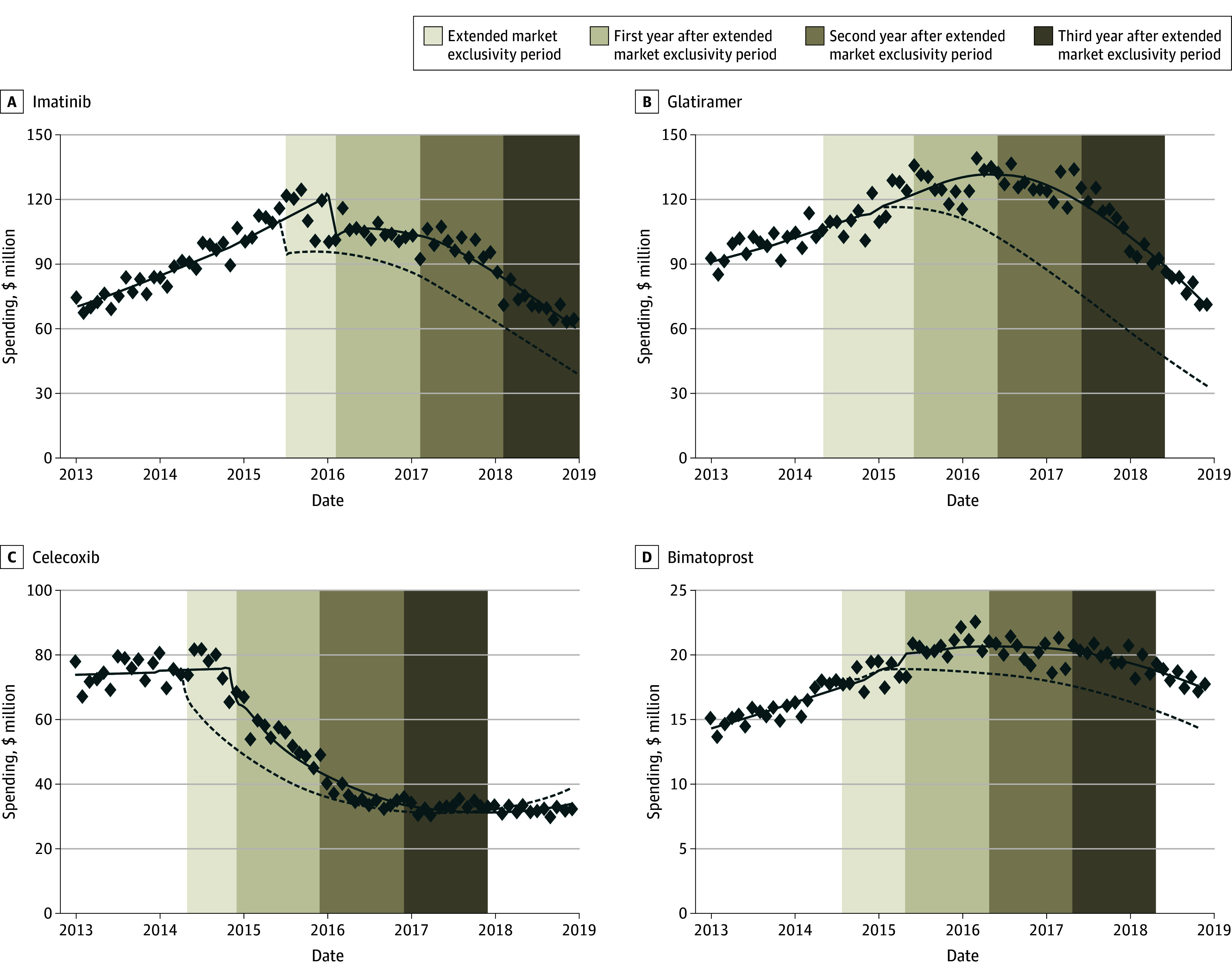
Estimated National Monthly Drug Spending With or Without Extended Market Exclusivity in Medicare The estimated monthly net spending for each drug in total Medicare plans. The estimates were weighted to extrapolate the spending estimates from the Medicare random sample to national levels, ensuring generalizability to the full Medicare Part D population. The solid line shows the estimated monthly spending of each drug in status quo scenario. The dashed line shows the estimated monthly spending of each drug in no extended exclusivity period. The data points are the actual monthly net spending. The area between the solid line and dashed line within the vertical area shows the excess spending of each drug in different time periods. Vertical axes are different for each drug. The excess spending is visually represented by the area between the solid and dashed lines within the shaded vertical areas. Medicare data are available only through 2018.

Spending in commercial plans would have decreased by $16 (95% CI, $2-$36) million for bimatoprost, $410 (95% CI, $280-$545) million for celecoxib, $421 (95% CI, $293-$561) million for imatinib, and $1.1 (95% CI, $0.5-$1.6) billion for glatiramer (Figure 2). In Medicare, spending would have decreased by $51 (95% CI, $10-$95) million for bimatoprost, $316 (95% CI, $143-$480) million for celecoxib, $586 (95% CI, $406-$769) million for imatinib, and $687 (95% CI, $228-$1115) million for glatiramer (Figure 3).

National spending would have decreased by $2.1 (95% CI, $1.4-$2.7) billion for the 1-year postextended exclusivity period and by $4.9 (95% CI, $4.1-$5.8) billion for the 3-year postextended exclusivity period (eTables 10-11 in Supplement 1). Most excess spending occurred in the first year after generic entry, with diminishing incremental savings in subsequent years (eTable 12 in Supplement 1). Across the 4 study drugs, cumulative excess spending as a percentage of pregeneric entry spending varied widely. By the 3-year postextended exclusivity period, excess spending accounted for 17.0% (bimatoprost) to 67.5% (glatiramer) of pregeneric entry spending in commercial plans and 35.8% (celecoxib) to 99.7% (glatiramer) in Medicare (eTable 13 in Supplement 1).

## Discussion

Generic entry for 4 top selling drugs (imatinib, glatiramer, celecoxib, and bimatoprost) was delayed by 7 to 13 months beyond expiration of the drugs’ key active ingredient patents. Over the full market exclusivity period and the 2 years following the actual start of generic competition, commercial plans spent $1.9 billion and Medicare spent $1.6 billion in excess costs that could have been avoided with timely generic competition. By 3 years postextended market exclusivity expiration, excess spending would have increased to $2.5 billion in commercial plans and $2.4 billion in Medicare, driven largely by imatinib and celecoxib. The excess spending was most pronounced in the first year following generic entry, when brand-name drugs still retained a considerable market share. Although possible cumulative savings from timely generic competition continued to grow over time, the incremental reduction in excess spending decreased in later years, reflecting the stabilization of generic competition. These findings suggest that most avoidable spending occurred early after delayed generic entry.

The number of prescriptions diverged between MarketScan and Medicare after generic entry. In commercial plans, the number of prescriptions increased postgeneric entry for all 4 drugs, suggesting broader access and substitution. However, in Medicare, the number of prescriptions declined for imatinib, glatiramer, and bimatoprost after generic entry. These differences may reflect plan-specific formulary management strategies. Future research could build on this analysis by exploring more drugs and further investigating price effects and shifts in utilization.

These results highlight the potential savings for payers and patients if policymakers limit the ability of brand-name manufacturers to strategically extend market exclusivity. Such interventions could serve the dual purpose of addressing excessive drug spending in the US and facilitating the entry of price-lowering generic competition that could help patients access needed medicines. Commercial plans, Medicare, and patients would benefit from savings with even slightly earlier generic competition for high-revenue drugs. For patients, this could lead to lower out-of-pocket costs and potentially reduce the need for premium increases in health insurance plans.

Savings for curbing market exclusivity extensions were highly variable. For example, bimatoprost was the lowest grossing of the 4 drugs in our sample. One explanation is that after generic drugs entered the market, generic bimatoprost was covered by only 35% of Medicare Part D plans, and the median out-of-pocket cost for patients using the generic version was approximately $30, compared with $45 for the branded version in 2017.^25^ Controversy over the interchangeability of brand-name vs generic ophthalmologic drug formulations was also likely a factor.^26^ Clinicians and payers can help mitigate the effects of market exclusivity extensions by ensuring that prescribers are more aware of the clinical interchangeability of FDA-approved generic drugs and that payer formularies favor FDA-approved lower-cost generic versions of brand-name products.^27,28,29^ Prior research also found that imatinib had poor generic competition, resulting in only limited price and spending reductions following the availability of generics.^30^ In our study, this pattern was reflected in both the level and slope changes, which were smaller compared with other drugs.

Policymakers can seek to address extensions of market exclusivity that delay generic competition beyond the expected patent expiration. Legislators and regulators at the US Patent and Trademark Office may seek approaches that limit manufacturers’ abilities to build the large patent portfolios that raise the complexity and cost of generic entry by ensuring that patents on new drug formulations or methods of use are properly granted and sufficiently innovative.^7,31^ Similarly, policymakers could improve access to patent reviews such as from the Patent Trial and Appeals Board, which could foster quicker generic competition, helping lower drug costs for payers and patients. Congressional legislation proposed in 2023 would have permitted brand-name drug manufacturers to assert 1 patent per group of closely related patents linked by a terminal disclaimer due to an obviousness-type double patenting rejection, promoting timely generic competition.^32^ Others have proposed establishing single, fixed exclusivity periods for all drugs, removing opportunities for extending exclusivity through additional patents on minor reformulations.

### Limitations

This retrospective serial cross-sectional study was subject to several limitations. First, the case study approach limited the generalizability of our results. We selected 4 top-selling drugs based on data availability and timing of generic entry. We focused on drugs with patent information comprehensively captured in the FDA’s Orange Book; and excluded biologic drugs because patents for biologic drugs are not disclosed in a similar comprehensive fashion. In addition, many potential study drugs lacked continuous rebate data in the SSR Health Database. As a result, our findings may not fully represent all drugs with extended market exclusivity. Another study estimated about $2 billion in excess spending from delayed market entry of biosimilar adalimumab to Medicare,^33^ suggesting variable savings from extending market exclusivity depending on the products chosen to analyze. By expressing excess spending as a percentage of pregeneric entry spending, we illustrate the relative financial burden associated with exclusivity extensions. The observed variation may be due to differences in market dynamics, the number of generic entrants, and payer-specific pricing policies. Future research could examine whether these patterns generalize across a broader set of drugs facing exclusivity extensions. Second, we did not evaluate the reasons for delayed generic competition for the study drugs, which may include factors such as insufficient generic competition^34,35^ and nonpatent-related delaying strategies.^15^ Third, we used SSR Health non-Medicaid discounts to estimate net spending for commercial plans and Medicare. These discounts include rebates, coupons, 340B Drug Discount Program prices, and price concessions to other federal purchasers. Accordingly, it is possible our estimates of net spending may be lower than actual net spending for commercial plans and Medicare.^36,37^ Finally, we did not assess excess spending in Medicaid because manufacturers must offer state Medicaid programs the best price associated with brand-name drugs in the private insurance market.^3^ The main exceptions to this rule include drugs sold to federal health systems such as the Department of Veterans Affairs and the Department of Defense, prices negotiated by private Medicare Part D plans, and discounts offered under the 340B Drug Pricing Program.^38^ In addition, manufacturers must provide additional rebates to Medicaid payers for price increases over time higher than the rate of inflation.^39^ All of the drugs in this study were on the market for extended periods of time during which they were subject to substantial price increases; for example, imatinib was initially priced at $26 000 per year when it was approved in 2011 but was available at $146 000 per year by 2017.^40^ As a result of inflation-linked rebates, Medicaid programs would be likely to pay low net prices for these products at the time of generic entry, potentially even lower than for the initial generic drugs, which do not have the same price increase history.

## Conclusions

This cross-sectional study of 4 top-selling prescription drugs found that extended brand-name drug market exclusivity contributes to excess spending in commercial plans and Medicare. Policies to limit the frequency and effects of such extensions by ensuring that competition from generic drugs occurs in a timely fashion as close to expiration of the patents on a drug’s active ingredient or other key innovation can help reducing excess spending on prescription drugs by patients and the health care system. Such approaches can help ensure that US patients do not experience unnecessary out-of-pocket spending and make more resources available for spending on other innovative prescription drugs.

## Notes

Supplement 1. eFigure. Flowchart of drug selection process for study cohort eMethod 1: Identification of the drug’s key patent eMethod 2. Gross cost measurements in each database eMethod 3. Weights for national spending estimates eMethod 4. Regression equations and confidence interval computations eTable 1. Reference and new formulation products in study cohort eTable 2. Linear time-series regression models for monthly spending in MarketScan sample eTable 3. Linear time-series regression models for monthly spending in Medicare sample eTable 4. Model fitness comparison eTable 5. Linear time-series regression models for monthly prescription volume in MarketScan sample eTable 6. Linear time-series regression models for monthly prescription volume in Medicare sample eTable 7. Excess spending associated with extended market exclusivity for 1-year generic competition in each database eTable 8. Excess spending associated with extended market exclusivity for 2-year generic competition in each database eTable 9. Excess spending associated with extended market exclusivity for 3-year generic competition in each database eTable 10. National excess spending associated with extended market exclusivity for 1-year generic competition eTable 11. National excess spending associated with extended market exclusivity for 3-year generic competition eTable 12. Estimated incremental and cumulative national excess spending eTable 13. Estimated cumulative excess spending as a percentage of pre-generic entry spending

Supplement 2. Data Sharing Statement
