# Supplementary material for: Estimating Costs of Market Exclusivity Extensions For 4 Top-Selling Prescription Drugs in the US
Source: JAMA Health Forum. 2025 Aug 22;6(8):e252631. doi: 10.1001/jamahealthforum.2025.2631 (PMC12374220; doi:10.1001/jamahealthforum.2025.2631)
Supplement: Supplement 1. — eFigure. Flowchart of drug selection process for study cohort eMethod 1: Identification of the drug’s key patent eMethod 2. Gross cost measurements in each database eMethod 3. Weights for national spending estimates eMethod 4. Regression equations and confidence interval computations eTable 1. Reference and new formulation products in study cohort eTable 2. Linear time-series regression models for monthly spending in MarketScan sample eTable 3. Linear time-series regression models for monthly spending in Medicare sample eTable 4. Model fitness comparison eTable 5. Linear time-series regression models for monthly prescription volume in MarketScan sample eTable 6. Linear time-series regression models for monthly prescription volume in Medicare sample eTable 7. Excess spending associated with extended market exclusivity for 1-year generic competition in each database eTable 8. Excess spending associated with extended market exclusivity for 2-year generic competition in each database eTable 9. Excess spending associated with extended market exclusivity for 3-year generic competition in each database eTable 10. National excess spending associated with extended market exclusivity for 1-year generic competition eTable 11. National excess spending associated with extended market exclusivity for 3-year generic competition eTable 12. Estimated incremental and cumulative national excess spending eTable 13. Estimated cumulative excess spending as a percentage of pre-generic entry spending [file jamahealthforum-e252631-s001.pdf]

Supplemental Online Content

Hong D, Tu SS, Beall RF, et al. Estimating costs of market exclusivity extensions for 4 top-selling prescription drugs in the US. *JAMA Health Forum*. 2025;6(8):e252631. doi:10.1001/jamahealthforum.2025.2631

eFigure. Flowchart of drug selection process for study cohort

eMethods 1. Identification of the drug's key patent

eMethods 2. Gross cost measurements in each database

eMethods 3. Weights for national spending estimates

eMethods 4. Regression equations and confidence interval computations

eTable 1. Reference and new formulation products in study cohort

eTable 2. Linear time-series regression models for monthly spending in MarketScan sample

eTable 3. Linear time-series regression models for monthly spending in Medicare sample

eTable 4. Model fitness comparison

eTable 5. Linear time-series regression models for monthly prescription volume in MarketScan sample

eTable 6. Linear time-series regression models for monthly prescription volume in Medicare sample

eTable 7. Excess spending associated with extended market exclusivity for 1-year generic competition in each database

eTable 8. Excess spending associated with extended market exclusivity for 2-year generic competition in each database

eTable 9. Excess spending associated with extended market exclusivity for 3-year generic competition in each database

eTable 10. National excess spending associated with extended market exclusivity for 1-year generic competition

eTable 11. National excess spending associated with extended market exclusivity for 3-year generic competition

eTable 12. Estimated incremental and cumulative national excess spending

eTable 13. Estimated cumulative excess spending as a percentage of pre-generic entry spending

© 2025 Hong D et al. *JAMA Health Forum*.

This supplemental material has been provided by the authors to give readers additional information about their work.

eFigure 1. Flowchart of drug selection process for study cohort

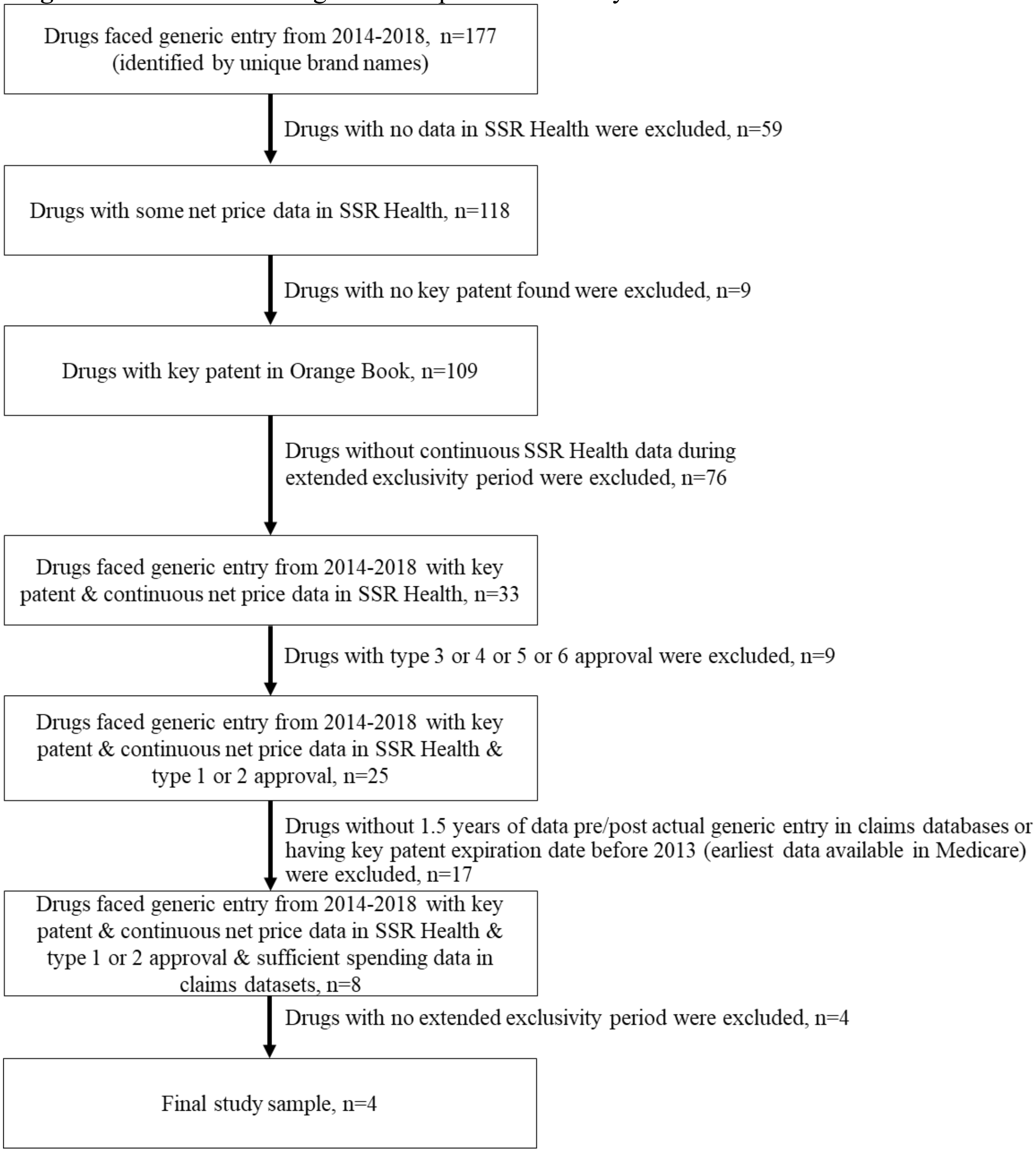

© 2025 Hong D et al. *JAMA Health Forum*.

© 2025 Hong D et al. *JAMA Health Forum*.

In our study, we systematically identified and categorized pharmaceuticals using patent information from the US Food and Drug Administration's (FDA's) Approved Drug Products with Therapeutic Equivalence Evaluations ("Orange Book"). We excluded biologic products due to incomplete public listing of their patents. The "key patents" were defined as the patents used to calculate the expected patent expiration date and extended market exclusivity. We categorized drugs into two groups: (1) those with patent term extensions (PTE) and (2) those without PTE.

(1) Drugs with PTE

The key patent was the patent with PTE. We identified the original expiration date (key patent) and the period of extension granted according to the list of patent terms extended under 35 U.S.C. § 156 from United States Patent and Trademark Office (USPTO). (https://www.uspto.gov/patents/laws/patent-term-extension/patent-terms-extended-under-35-usc-156)

(2) Drugs without PTE

We first identified whether the drug's first patent was filed before the General Agreement on Tariffs and Trade (GATT). Before GATT, US patents were protected for 17 years from the date of issuance. Post-GATT, the term of protection starts from the date of the grant of the patent and lasts 20 years from the application filing date. This change was effective from June 8, 1995.

If the drug's first patent was filed before GATT, we reviewed all the patents of the drug, with the key patent defined as the patent with composition claims. If there were multiple patents with composition claims, the patent with broadest claims was considered the key patent. If there was no patent with composition claims, the drug was excluded as lacking a key patent. If the drug's first patent was filed after GATT, the key patent was the drug's first filed patent listed in the Orange Book.

eMethod 2. Gross cost measurements in each database

1. MarketScan

Prescription drug spending (gross costs) = payer's costs (payer's net payment + coordination of benefit and other savings) + patient's out-of-pocket costs (patient's copayment + coinsurance + deductible)

2. Medicare

Prescription drug spending (gross costs) = the gross drug costs recorded in claims

eMethod 3. Weights for national spending estimates

To estimate net spending on the four drugs among the full commercially insured and Medicare populations in the US, we applied weights. The weights are used to make the sample (MarketScan beneficiaries/Medicare random sample) more representative of the target population (US commercially insured population/total beneficiaries in Medicare Part D). Each weight is described below.

- The weight for commercially insured population = number of beneficiaries in US commercially insured population/ in MarketScan in each year
- The weight for Medicare population = number of beneficiaries in Medicare Part D / Medicare random sample in each year

© 2025 Hong D et al. *JAMA Health Forum*.

Note: The number of beneficiaries in the U.S. commercially insured population is the sum of individuals with 'Employer' and 'Non-Group' coverage, as reported by the Kaiser Family Foundation.

This adjustment was made to ensure that our estimates reflect the broader population, accounting for the sample's structure or distribution. By applying these weights in our regression model, we adjusted for sample variability and ensured that the regression model gave greater influence on observations with higher weights, making our spending estimates more reliable within the constraints of our sample. After obtaining predicted costs, we multiplied each by the corresponding weight to scale the estimates to each broader target population for each year and month. This step allows each prediction to be adjusted upward to reflect what spending would look like at a national level in each insurance type, based on our sample data. For each month and year, each estimated cost of the target population is described below.

- The national predicted cost for commercially insured population = predicated cost in Marketscan ×analytic weight for commercially insured population
- The national predicted cost for national Medicare = predicated cost in Marketscan × analytic weight for national Medicare

eMethod 4. Regression equations and confidence interval computations
We used segmented linear regression to estimate the impact of extended market exclusivity on drug spending and prescription volume, applying an interrupted time series approach with monthly spending data. For each drug, we modeled spending trajectories under the status quo scenario (extended exclusivity remains in place) and compared it to a counterfactual scenario (assuming generic entry at the key patent expiration date).

The model is specified as follows:

$$\log(Y_t) = \beta_0 + \beta_1 Time_t + \beta_2 Postperiod_t + \beta_3 Time_t \times Postperiod_t + \varepsilon_t$$

<u>Where:</u>
Y_t = Monthly total net spending or monthly number of prescriptions filled. We did not use log transformation for the number of prescriptions filled.
β_0= Baseline level (log-transformed monthly spending or monthly total prescription volume) at the start of the study period
β_1 = Baseline slope (log-transformed monthly spending trend or monthly prescription volume trend before generic entry,)
$Time_t$ = Continuous time variable (number of months since the start of the study)
β_2 = Immediate level change at the time of generic entry (captures sudden shifts in log-transformed spending or prescription volume)
$Postperiod_t$ = Binary indicator (0 before generic entry, 1 after generic entry)
β_3 = Slope change (change in trend post-generic entry)
$Time_t \times Postperiod_t$ = Interaction term (modifies the post-generic trend)
ε_t = Error term, accounting for serial correlation if present

For drugs in Medicare random sample, we included a quadratic post-period term when goodness-of-fit measures (e.g., adjusted R^2) indicated improved model performance (i.e., adjusted R^2 increased at least 0.03 compared to the model without quadratic post-period term):

© 2025 Hong D et al. *JAMA Health Forum*.

$$Y_t = \beta_0 + \beta_1 Time_t + \beta_2 Postperiod_t + \beta_3 Time_t \times Postperiod_t + \beta_4 (Time_t \times Postperiod_t)^2 + \varepsilon_t$$

To estimate national excess spending, we followed these steps:

1. Modeled spending trajectories under the status quo scenario (extended exclusivity remains in place) using segmented regression on log-transformed monthly spending and applying these weights in our regression models.
2. Estimated counterfactual spending by applying the pre-generic entry slope and level change coefficients at the key patent expiration date, rather than at the actual observed generic entry date.
3. Exponentiated the predicted log-spending for each scenario in both MarketScan and Medicare samples to obtain estimated spending in real dollar terms.
4. Applied weights to extrapolate the spending estimates from each sample to national levels, ensuring generalizability to the full commercially insured and Medicare Part D populations.
5. Calculated excess spending as the difference between the two scenarios over the extended exclusivity period plus 1, 2, and 3 years post-generic entry.

To compute confidence intervals for the excess spending in points 5. we used parametric bootstrap approach coded in R: We regenerated 5,000 times the logarithm of spending trajectory from a Gaussian distribution centered the fitted model under the status quo, estimating the variance from the residuals (with homoscedasticity assumption). For each generated spending trajectory, we applied steps 1. to 5. Finally, the lower and upper limits of the CI have been set to the 2.5% and 97.5% quantiles of the bootstrap distributions for the estimates in Step 5. We used the same approach for the total spending (combining commercial and Medicare), computing quantiles of the bootstrap distributions of the corresponding totals.

© 2025 Hong D et al. *JAMA Health Forum*.

Table A1. Reference and new formulation products in study cohort

Drug Name	Original Indication[a]	Product Category	Approval Date	Product Change Category	Product Characteristics	Discontinued Year	Key Patent Expiration Date	Date of First Generic Entry
Imatinib (Gleevec)	Cancer	Reference	05/10/2001	N/A	Capsule, 50mg, 100mg	2010[b]	07/04/2015	None
		New formulation #1	04/18/2003	New dose form, strength	Tablet, 100 mg 400 mg			02/2016
Glatiramer (Copaxone)	Multiple Sclerosis	Reference	12/20/1996	N/A	Solution, subcutaneous	2010	05/24/2014	None
		New formulation #1	02/12/2002	New dose form	Injectable, subcutaneous 20mg/ml			06/2015
		New formulation #2	01/28/2014	New dose form, strength	Injectable, subcutaneous 40mg/ml			10/2017
Celecoxib (Celebrex)	Arthritis	Reference	12/31/1998	N/A	Capsule, 100mg, 200mg		05/30/2014	12/2014
		New formulation #1	08/29/2002	New dose form, strength	Capsule, 400 mg			12/2014
		New formulation #2	12/15/2006	New dose form, strength	Capsule, 50 mg			12/2014
Bimatoprost (Lumigan)	Glaucoma	Reference	03/16/2001	N/A	Solution/drops, ophthalmic 0.03%	2014[b]	08/20/2014	05/2015
		New formulation #1	08/31/2010	New dose strength	Solution/drops, ophthalmic 0.01%			None

Notes: [a] All new formulation products included the original indication; [b] Federal Register determination that product was not discontinued or withdrawn for safety or effectiveness reasons.

© 2025 Hong D et al. *JAMA Health Forum*.

Table A2. Linear time-series regression models for monthly spending in MarketScan sample

	Coefficient Estimates (95% CI) [a]			
	**Imatinib (Gleevec)**	**Glatiramer (Copaxone)**	**Celecoxib (Celebrex)**	**Bimatoprost (Lumigan)**
Baseline level (log scale)	3.18 (3.13, 3.23) ***	3.99 (3.92, 4.06) ***	3.14 (3.05, 3.24) ***	1.13 (1.06, 1.20) ***
Baseline level (exponentiated, $ in million)	24.10 (23.15, 25.08) ***	53.96 (51.67, 56.34) ***	23.13 (22.33, 23.94) ***	3.09 (2.73, 3.49) ***
Baseline slope (log scale)	8.85×10^{-4} (-5.77×10^{-4}, 2.35×10^{-3})	-4.12×10^{-3} (-6.26×10^{-3}, -1.98×10^{-3}) ***	-3.86×10^{-3} (-7.26×10^{-3}, -4.55×10^{-4}) **	-6.86×10^{-3} (-9.03×10^{-3}, -4.70×10^{-3}) ***
Baseline slope (exponentiated)	1.00 (1.00, 1.00)	1.00 (0.99, 1.00) ***	1.00 (0.99, 1.00) **	0.99 (0.99, 1.00) ***
Change in level (log scale)	-7.12×10^{-2} (-0.13, -1.24×10^{-3}) ***	0.12 (5.10×10^{-2}, 0.20) **	-0.52 (-0.62, -0.42) ***	-9.97×10^{-2} (-0.17, 2.81×10^{-2}) **
Change in level (exponentiated	0.93 (0.87, 1.00) ***	1.13 (1.03, 1.24) **	0.60 (0.53, 0.68) ***	0.91 (0.80, 1.03) **
Change in slope (log scale)	-1.72×10^{-2} (-1.89×10^{-2}, -1.55×10^{-2}) ***	-1.86×10^{-2} (-2.09×10^{-2}, -1.63×10^{-2}) ***	-2.16×10^{-2} (-2.51×10^{-2}, -1.80×10^{-2}) ***	-3.30×10^{-3} (-5.61×10^{-3}, -9.80×10^{-4}) ***
Change in slope (exponentiated)	0.98 (0.98, 0.98) ***	0.98 (0.98, 0.98) ***	0.98 (0.98, 0.98) ***	1.00 (0.99, 1.00) ***

[a] The table shows the output from the linear time-series models of the monthly total net spending from January 2011 to December 2020 using MarketScan data. Costs were log-transformed for regression analyses. CI = confidence interval.

Note: No quadratic post-period term was included in these models.

© 2025 Hong D et al. *JAMA Health Forum*.

Table A3. Linear time-series regression models for monthly spending in Medicare sample

	Coefficient Estimates (95% CI) [a]			
	**Imatinib (Gleevec)**	**Glatiramer (Copaxone)**	**Celecoxib (Celebrex)**	**Bimatoprost (Lumigan)**
Baseline level (log scale)	2.16 (2.12, 2.20) ***	2.42 (2.38, 2.46) ***	2.21 (2.15, 2.26) ***	0.57 (0.53, 0.60) ***
Baseline level (exponentiated, $ in million)	8.67 (8.32, 9.02) ***	11.23 (10.79, 11.68) ***	9.09 (8.62, 9.58) ***	1.76 (1.71, 1.82) ***
Baseline slope (log scale)	1.44×10^{-2} (1.25×10^{-2}, 1.63×10^{-2}) ***	9.17×10^{-3} (6.75×10^{-3}, 1.16×10^{-2}) ***	6.36×10^{-4} (-3.45×10^{-3}, 4.72×10^{-3})	1.01×10^{-2} (8.12×10^{-3}, 1.21×10^{-2}) ***
Baseline slope (exponentiated)	1.01 (1.01, 1.02) ***	1.01 (1.01, 1.01) ***	1.00 (1.00, 1.00)	1.01 (1.01, 1.01) ***
Change in level (log scale)	-0.16 (-0.23, -8.77×10^{-2}) ***	-1.44×10^{-3} (-6.43×10^{-2}, 6.14×10^{-2})	-0.14 (-0.21, -5.98×10^{-2}) **	3.20×10^{-2} (-1.76×10^{-2}, 8.15×10^{-2})
Change in level (exponentiated	0.85 (0.79, 0.92) ***	1.00 (0.94, 1.06)	0.87 (0.81, 0.94) **	1.03 (0.98, 1.08)
Change in slope (log scale)	-8.76×10^{-3} (-1.71×10^{-2}, -4.21×10^{-4}) ***	4.76×10^{-3} (-9.76×10^{-4}, 1.05×10^{-2})	-4.36×10^{-2} (-5.02×10^{-2}, -3.71×10^{-2}) ***	-5.93×10^{-3} (-1.04×10^{-2}, -1.50×10^{-3}) **
Change in slope (exponentiated)	0.99 (0.98, 1.00) ***	1.00 (1.00, 1.01)	0.96 (0.95, 0.96) ***	0.99 (0.99, 1.00) **
Change in square of slope (log scale) [b]	-6.45×10^{-4} (-8.75×10^{-4}, -4.14×10^{-4}) *	-6.43×10^{-4} (-7.63×10^{-4}, -5.24×10^{-4}) ***	5.97×10^{-4} (4.94×10^{-4}, 7.00×10^{-4}) ***	-1.79×10^{-4} (-2.67×10^{-4}, -8.99×10^{-5}) ***
Change in square of slope (exponentiated)	1.00 (1.00, 1.00) *	1.00 (1.00, 1.00) ***	1.00 (1.00, 1.00) ***	1.00 (1.00, 1.00) ***

[a] The table shows the output from the linear time-series models of the monthly total net spending from January 2013 to December 2018 using Medicare data. Costs were log-transformed for regression analyses. CI = confidence interval.

[b] Change in square of slope indicates the square of interaction between time and post period. This variable was added when the models had better goodness-of-fit with larger R-squares than models without this variable.

* P < .05 ** P < .01 *** P < .001

© 2025 Hong D et al. *JAMA Health Forum*.

Table A4. Model fitness comparison

	Sample	Model Specification	R^2	Adjusted R^2	Decision
Imatinib (Gleevec)	Medicare	Linear model (no quadratic)	0.8287	0.8212	Not selected
		Linear + quadratic term	0.8831	0.8761	Selected
	MarketScan	Linear model (no quadratic)	0.9616	0.9607	Selected
		Linear + quadratic term	0.9631	0.9619	Not selected
Glatiramer (Copaxone)	Medicare	Linear model (no quadratic)	0.7154	0.7028	Not selected
		Linear + quadratic term	0.8954	0.8891	Selected
	MarketScan	Linear model (no quadratic)	0.9758	0.9752	Selected
		Linear + quadratic term	0.9763	0.9756	Not selected
Celecoxib (Celebrex)	Medicare	Linear model (no quadratic)	0.9195	0.9160	Not selected
		Linear + quadratic term	0.9732	0.9716	Selected
	MarketScan	Linear model (no quadratic)	0.9821	0.9817	Selected
		Linear + quadratic term	0.9908	0.9905	Not selected
Bimatoprost (Lumigan)	Medicare	Linear model (no quadratic)	0.8204	0.8124	Not selected
		Linear + quadratic term	0.8552	0.8466	Selected
	MarketScan	Linear model (no quadratic)	0.9398	0.9384	Selected
		Linear + quadratic term	0.9406	0.9387	Not selected

Table A5. Linear time-series regression models for monthly prescription volume in MarketScan sample

	Coefficient Estimates (95% CI) [a]			
	Imatinib (Gleevec)	Glatiramer (Copaxone)	Celecoxib (Celebrex)	Bimatoprost (Lumigan)
Baseline level	2793.69 (2715.12, 2872.26) ***	10311.20 (9814.28, 10808.12) ***	100838.60 (97404.22, 104273.00) ***	30196.97 (28358.96, 32034.97) ***
Baseline slope	-19.63 (-21.80, -17.46) ***	-90.69 (-105.40, -75.98) ***	-906.91 (-1039.68, -774.14) ***	-257.42 (-320.68, -194.15) ***
Change in level	83.38 (-14.59, 181.35)	332.00 (-117.54, 781.53)	-6307.99 (-11203.68, -1412.30) *	-4031.80 (-6020.93, -2042.67) ***
Change in slope	13.30 (10.87, 15.74) ***	46.13 (31.28, 60.97) ***	745.91 (601.31, 890.52) ***	180.26 (115.79, 244.74) ***

[a] The table shows the output from the linear time-series models of monthly prescription volume from January 2011 to December 2020 using MarketScan data. CI = confidence interval.

* P < .05 ** P < .01 *** P < .001

Table A6. Linear time-series regression models for monthly prescription volume in Medicare sample

	Coefficient Estimates (95% CI) [a]			
	Imatinib (Gleevec)	Glatiramer (Copaxone)	Celecoxib (Celebrex)	Bimatoprost (Lumigan)
Baseline level	1030.20 (1000.71, 1059.69) ***	2105.61 (2023.60, 2187.63) ***	36969.87 (35675.91, 38263.83) ***	18404.81 (17628.08, 19181.54) ***
Baseline slope	3.94 (2.62, 5.25) ***	6.36 (1.06, 11.66) *	-149.91 (-237.62, -62.19) ***	41.81 (-11.78, 95.39)
Change in level	32.34 (-0.59, 65.28)	297.15 (167.50, 426.80) ***	-2193.81 (-3570.79, -816.82) **	-347.81 (-1230.70, 535.09)
Change in slope	-7.66 (-9.60, -5.71) ***	-15.50 (-21.64, -9.37) ***	179.28 (89.59, 268.97) ***	-122.47 (-178.07, -66.87) ***

[a] The table shows the output from the linear time-series models of monthly prescription volume from January 2013 to December 2018 using Medicare data. CI = confidence interval.
* P < .05 ** P < .01 *** P < .001

Table A7. Excess spending associated with extended market exclusivity for 1-year generic competition in each database

	**Estimated spending, US $ (in million, 95% CI)**					
	MarketScan			Medicare Random Sample		
	With extended market exclusivity	Without extended market exclusivity	Excess spending	With extended market exclusivity	Without extended market exclusivity	Excess spending
Imatinib (Gleevec)	608 (575, 643)	556 (527, 587)	52 (29, 75)	261 (244, 281)	221 (203, 242)	40 (21, 60)
Glatiramer (Copaxone)	1139 (1070, 1216)	1028 (941, 1128)	111 (9, 218)	379 (358, 401)	351 (316, 390)	28 (-16, 70)
Celecoxib (Celebrex)	267 (243, 292)	188 (169, 210)	79 (51, 106)	148 (138, 160)	117 (104, 131)	32 (16, 48)
Bimatoprost (Lumigan)	44 (41, 47)	41 (38, 45)	3 (-1, 6)	52 (50, 54)	49 (46, 53)	3 (-1, 6)
Total	2058.4	1813.6	244.8	840.7	738.4	102.3

CI = confidence interval

© 2025 Hong D et al. *JAMA Health Forum*.

Table A8. Excess spending associated with extended market exclusivity for 2-year generic competition in each database

	Estimated spending, US $ (in million, 95% CI)					
	MarketScan			Medicare Random Sample		
	With extended market exclusivity	Without extended market exclusivity	Excess spending	With extended market exclusivity	Without extended market exclusivity	Excess spending
Imatinib (Gleevec)	819 (777, 861)	743 (706, 783)	76 (50, 102)	394 (371, 419)	325 (302, 351)	69 (47, 91)
Glatiramer (Copaxone)	1527 (1440, 1621)	1332 (1226, 1451)	195 (81, 313)	560 (534, 587)	478 (432, 529)	82 (27, 135)
Celecoxib (Celebrex)	354 (325, 384)	263 (238, 290)	91 (61, 121)	202 (190, 216)	164 (148, 183)	38 (18, 58)
Bimatoprost (Lumigan)	64 (60, 67)	60 (56, 65)	3 (-1, 7)	81 (78, 85)	75 (71, 80)	6 (1, 11)
Total	2763.2	2398.5	364.8	1238.7	1042.5	196.2

CI = confidence interval

Table A9. Excess spending associated with extended market exclusivity for 3-year generic competition in each database

Estimated spending, US $ (in million, 95% CI)						
	MarketScan			Medicare Random Sample		
	With extended market exclusivity	Without extended market exclusivity	Excess spending	With extended market exclusivity	Without extended market exclusivity	Excess spending
Imatinib (Gleevec)	992 (945, 1040)	897 (856, 941)	96 (67, 125)	495 (469, 522)	398 (371, 429)	97 (71, 123)
Glatiramer (Copaxone)	1822 (1724, 1927)	1564 (1447, 1696)	258 (133, 387)	707 (678, 738)	563 (509, 622)	144 (80, 205)
Celecoxib (Celebrex)	418 (387, 452)	318 (290, 349)	100 (68, 132)	248 (235, 262)	209 (189, 231)	39 (15, 62)
Bimatoprost (Lumigan)	81 (76, 85)	77 (72, 82)	4 (-1, 8)	109 (105, 113)	99 (93, 105)	10 (4, 16)
Total	3313.0	2855.7	457.3	1557.2	1263.8	293.4

CI = confidence interval

© 2025 Hong D et al. *JAMA Health Forum*.

Table A10. national excess spending associated with extended market exclusivity for 1-year generic competition

Estimated national spending, US $ (in million, 95% CI)							
	Commercial			Medicare			National Estimates
	With extended market exclusivity	Without extended market exclusivity	Excess spending	With extended market exclusivity	Without extended market exclusivity	Excess spending	Total Excess spending
Imatinib (Gleevec)	3121 (2957, 3299)	2845 (2696, 3003)	276 (162, 401)	2185 (2046, 2341)	1854 (1706, 2016)	331 (178, 484)	607 (419, 803)
Glatiramer (Copaxone)	5394 (5063, 5746)	4816 (4408, 5284)	578 (96, 1059)	3149 (2981, 3332)	2913 (2631, 3232)	236 (-137, 582)	814 (209, 1403)
Celecoxib (Celebrex)	1201 (1100, 1313)	857 (767, 956)	344 (230, 464)	1229 (1141, 1329)	966 (863, 1084)	264 (125, 397)	608 (431, 788)
Bimatoprost (Lumigan)	210 (198, 224)	197 (183, 214)	13 (-2, 29)	432 (413, 454)	410 (381, 439)	22 (-9, 55)	35 (0, 72)
Total	9927 (9534, 10336)	8715 (8259, 9200)	1211 (698, 1713)	6995 (6759, 7254)	6143 (5804, 6530)	852 (429, 1261)	2064 (1418, 2731)

CI = confidence interval

Table A11. national excess spending associated with extended market exclusivity for 3-year generic competition

Estimated national spending, US $ (in million, 95% CI)							
	Commercial			Medicare			National Estimates
	With extended market exclusivity	Without extended market exclusivity	Excess spending	With extended market exclusivity	Without extended market exclusivity	Excess spending	Total Excess spending
Imatinib (Gleevec)	5534 (5279, 5802)	4985 (4755, 5226)	549 (405, 707)	4180 (3978, 4407)	3327 (3103, 3580)	854 (639, 1064)	1403 (1136, 1665)
Glatiramer (Copaxone)	9444 (8929, 9977)	7995 (7397, 8662)	1449 (824, 2064)	5904 (5670, 6154)	4693 (4249, 5185)	1211 (686, 1705)	2659 (1862, 3451)
Celecoxib (Celebrex)	2053 (1905, 2211)	1589 (1450, 1742)	464 (321, 611)	2064 (1953, 2184)	1740 (1582, 1925)	324 (119, 516)	788 (543, 1030)
Bimatoprost (Lumigan)	427 (405, 451)	408 (382, 436)	19 (-2, 42)	913 (883, 946)	826 (775, 877)	87 (35, 141)	106 (50, 164)
Total	17457 (16872, 18066)	14977 (14311, 15695)	2481 (1826, 3143)	13061 (12737, 13427)	10586 (10060, 11174)	2475 (1877, 3049)	4956 (4074, 5832)

CI = confidence interval

© 2025 Hong D et al. *JAMA Health Forum*.

Table A12. Estimated incremental and cumulative national excess spending

Drug	Population	Year 1 Incremental Excess Spending ($ in million)	Year 2 Incremental Excess Spending ($ in million)	Year 3 Incremental Excess Spending ($ in million)	Cumulative Spending (1 Year, $ in million)	Cumulative Spending (2 Years, $ in million)	Cumulative Spending (3 Years, $ in million)
Imatinib (Gleevec)	Commercial	276	145	129	276	421	549
	Medicare	331	255	254	331	587	841
Glatiramer Acetate (Copaxone)	Commercial	578	475	396	578	1053	1449
	Medicare	236	451	524	236	687	1211
Celecoxib (Celebrex)	Commercial	344	66	54	344	410	464
	Medicare	264	52	8	264	316	324
Bimatoprost (Lumigan)	Commercial	13	3	3	13	16	20
	Medicare	22	29	36	22	51	87

Table A13. Estimated cumulative excess spending as a percentage of pre-generic entry spending

Drug	Population	Total Spending in Year Prior to Generic Entry ($ in million)	1-Year Excess Spending (% of Pre-Generic Entry Spending)	2-Year Excess Spending (% of Pre-Generic Entry Spending)	3-Year Excess Spending (% of Pre-Generic Entry Spending)	Cumulative Excess Spending (1 Year, $ in million)	Cumulative Excess Spending (2 Years, $ in million)	Cumulative Excess Spending (3 Years, $ in million)
Imatinib (Gleevec)	Commercial	1413	19.5%	29.8%	38.9%	276	421	549
	Medicare	1246	26.6%	47.1%	67.5%	331	587	841
Glatiramer (Copaxone)	Commercial	2146	26.9%	49.1%	67.5%	578	1053	1449
	Medicare	1215	19.4%	56.6%	99.7%	236	687	1211
Celecoxib (Celebrex)	Commercial	896	38.4%	45.8%	51.7%	344	410	464
	Medicare	905	29.1%	34.9%	35.8%	264	316	324
Bimatoprost (Lumigan)	Commercial	115	11.4%	14.2%	17.0%	13	16	20
	Medicare	197	11.0%	25.9%	44.0%	22	51	87

Note: Pre-generic entry spending was calculated as the total observed spending in the 12 months preceding the generic entry date, derived from claims data in MarketScan and Medicare random samples. These values were then scaled to the national level using analytic weights, as described in the Methods section. Excess spending percentages were calculated as the ratio of cumulative excess spending in each post-generic entry year to pre-generic entry spending.

© 2025 Hong D et al. *JAMA Health Forum*.
