# Supplementary material for: Estimating Costs of Market Exclusivity Extensions For 4 Top-Selling Prescription Drugs in the US
Source: JAMA Health Forum. 2025 Aug 22;6(8):e252631. doi: 10.1001/jamahealthforum.2025.2631 (PMC12374220; doi:10.1001/jamahealthforum.2025.2631)
Supplement: Supplement 2. — Data Sharing Statement [file jamahealthforum-e252631-s002.pdf]

Data Sharing Statement

Hong. Estimating Costs of Market Exclusivity Extensions For 4 Top-Selling Prescription Drugs in the US. *JAMA Health Forum*. Published August 22, 2025.
doi:10.1001/jamahealthforum.2025.2631

Data
Data available: Yes
Data types: Data (not involving human participants), Data dictionary
How to access data: Study data will be made available to researchers who submit a request.
When available: With publication

Supporting Documents
Document types: None

Additional Information
Who can access the data: Researchers whose proposed use of the data has been approved.
Types of analyses: For any purpose deemed to have academic value.
Mechanisms of data availability: After approval of a proposal.
Any additional restrictions: None.
